# Observational study of population genomic screening for variants associated with endocrine tumor syndromes in a large, healthcare-based cohort

**DOI:** 10.1186/s12916-022-02375-4

**Published:** 2022-06-07

**Authors:** Juliann M. Savatt, Nicole M. Ortiz, Gretchen M. Thone, Whitney S. McDonald, Melissa A. Kelly, Alexander S. F. Berry, Madiha M. Alvi, Miranda L. G. Hallquist, Jennifer Malinowski, Nicholas C. Purdy, Marc S. Williams, Amy C. Sturm, Adam H. Buchanan

**Affiliations:** 1 Genomic Medicine Institute, Geisinger, Danville, PA USA; 2 Autism & Developmental Medicine Institute, Geisinger, Lewisburg, PA USA; 3 Endocrinology, Diabetes, and Metabolism, Geisinger, Danville, PA USA; 4 Geisinger Commonwealth School of Medicine, Scranton, PA USA; 5 Otolaryngology, Geisinger, Danville, PA USA; 6 Heart and Vascular Institute, Geisinger, Danville, PA USA

**Keywords:** MEN 1, MEN 2, VHL, Succinate dehydrogenase, Genomic screening, Medullary thyroid cancer, Paraganglioma

## Abstract

**Background:**

In current care, patients’ personal and self-reported family histories are primarily used to determine whether genetic testing for hereditary endocrine tumor syndromes (ETS) is indicated. Population genomic screening for other conditions has increased ascertainment of individuals with pathogenic/likely pathogenic (P/LP) variants, leading to improved management and earlier diagnoses. It is unknown whether such benefits occur when screening broader populations for P/LP ETS variants. This manuscript assesses clinical utility outcomes of a large, unselected, healthcare-based genomic screening program by describing personal and family history of syndrome-related features, risk management behaviors after result disclosure, and rates of relevant post-disclosure diagnoses in patient-participants with P/LP ETS variants.

**Methods:**

Observational study of individuals informed of a P/LP variant in *MEN1*, *RET*, *SDHAF2*, *SDHB*, *SDHC*, *SDHD*, or *VHL* through Geisinger’s MyCode Community Health Initiative between June 2016 and October 2019. Electronic health records (EHRs) of participants were evaluated for a report of pre-disclosure personal and self-reported family histories and post-disclosure risk management and diagnoses.

**Results:**

P/LP variants in genes of interest were identified in 199 of 130,490 (1 in 656) adult Geisinger MyCode patient-participants, 80 of which were disclosed during the study period. Eighty-one percent (*n* = 65) did not have prior evidence of the result in their EHR and, because they were identified via MyCode, were included in further analyses. Five participants identified via MyCode (8%) had a personal history of syndrome-related features; 16 (25%) had a positive self-reported family history. Time from result disclosure to EHR review was a median of 0.7 years. Post-disclosure, 36 (55.4%) completed a recommended risk management behavior; 11 (17%) were diagnosed with a syndrome-related neoplasm after completing a risk management intervention.

**Conclusions:**

Broader screening for pathogenic/likely pathogenic variants associated with endocrine tumor syndromes enables detection of at-risk individuals, leads to the uptake of risk management, and facilitates relevant diagnoses. Further research will be necessary to continue to determine the clinical utility of screening diverse, unselected populations for such variants.

**Supplementary Information:**

The online version contains supplementary material available at 10.1186/s12916-022-02375-4.

## Background

Several autosomal dominant, hereditary syndromes are associated with an increased risk of developing tumors of the endocrine and neuroendocrine system, including multiple endocrine neoplasia type 1 (MEN 1; OMIM 131100), multiple endocrine neoplasia type 2 (MEN 2; OMIM 155240, 171400, 155240), hereditary paraganglioma and pheochromocytoma (PGL/PCC; OMIM 115310, 605373, 601650, 168000), and von Hippel-Lindau (VHL; OMIM 193300) syndromes [1]. These conditions are considered rare disorders, with a collective disease prevalence of approximately 1 in 8500 [2].

MEN 1 is caused by heterozygous, loss-of-function variants in the *MEN1* tumor suppressor gene (OMIM 613733) and is associated with endocrine and non-endocrine features. Individuals with MEN 1 have an increased risk of developing parathyroid gland hyperplasia, pituitary neoplasms, pancreatic neuroendocrine tumors, and, less commonly, adrenocortical tumors, lipomas, angiofibromas, collagenomas, meningiomas, ependymomas, schwannomas, leiomyomas, and leiomyosarcomas [3, 4].

MEN 2 is caused by missense, heterozygous, gain-of-function variants in the *RET* protooncogene (OMIM 164761) and is associated with an increased risk for medullary thyroid cancer. MEN 2 includes two clinically defined subtypes—MEN 2A and MEN 2B. MEN 2A can be further subdivided into classical MEN 2A, MEN 2A with cutaneous lichen amyloidosis (MEN 2A and CLA), MEN 2A and Hirschsprung disease (MEN 2A and HD), and familial medullary thyroid cancer (FMTC). Depending on the MEN 2A subtype, individuals might be at an increased risk for pheochromocytoma, primary hyperparathyroidism, cutaneous lichen amyloidosis, and/or Hirschsprung disease [5]. MEN 2B is associated with an earlier onset of MTC and pheochromocytomas compared to MEN 2A and may present with additional physical features including mucosal neuromas, intestinal ganglioneuromatosis, and characteristic physical features. Associations between MEN 2 subtype, aggressiveness of MTC, and the protein codon impacted by the DNA variant have been established [5].

PGL/PCC syndromes are caused by heterozygous, loss-of-function variants in genes coding for proteins involved in the mitochondrial respiratory chain complex II, including, but not limited to, *SDHAF2* (OMIM 613019), *SDHB* (OMIM 185470), *SDHC* (OMIM 602413), and *SDHD* (OMIM 602690) (hereafter referred to as “*SDHx*” genes)*.* Individuals with PGL/PCC syndromes are at an increased risk of developing paragangliomas, pheochromocytomas, renal cell carcinoma, and gastrointestinal stromal tumors (GISTs) [6–8]. Although heterozygous, pathogenic/likely pathogenic (P/LP) variants in these *SDHx* genes increase the risk for paragangliomas, the location of these tumors, biochemical phenotype, and risk for malignancy have been correlated with the gene impacted [9]. Additionally, *SDHAF2* and *SDHD* exhibit a parent-of-origin effect in which tumor development primarily occurs in individuals that inherit the variant on the paternal allele [10]. Homozygous and compound heterozygous P/LP variants in *SDHx* genes are associated with biochemical disorders such as the mitochondrial complex II deficiencies [11]. Since these are severe, early-onset disorders that usually are diagnosed clinically, they are not considered in this study.

Finally, VHL syndrome is caused by heterozygous, loss-of-function variants in the *VHL* tumor suppressor gene (OMIM 602690). Individuals with such variants are at an increased risk for hemangioblastomas of the brain, spinal cord, and retina; renal cell carcinoma; pheochromocytomas; paragangliomas; pancreatic neuroendocrine tumors; and papillary cystadenomas of the epididymis and broad ligament [12].

It is recommended that individuals with P/LP variants in *MEN1*, *RET*, an *SDHx* gene, or *VHL* undergo periodic biochemical and imaging surveillance for associated phenotypes [4, 9, 13–20]. Should endocrine tumors or other features be identified through this surveillance, additional symptomatic management may be indicated (e.g., hyperparathyroidism in a patient with MEN 1 may warrant parathyroidectomy). Additionally, prophylactic thyroidectomy is considered for individuals with P/LP *RET* variants [5, 18].

Germline genetic testing for these hereditary syndromes has historically been pursued when an individual’s personal and/or self-reported family history is suggestive of the condition (e.g., *RET* sequencing when a person is diagnosed with medullary thyroid cancer or *SDHx* testing after a paraganglioma of the head or neck is diagnosed), as the presence of a P/LP variant informs additional tumor risks, guides risk management, and enables testing of at-risk relatives [5, 21–24]. However, recent studies examining clinical genetic testing practices for other hereditary cancer syndromes and familial hypercholesterolemia have shown that indication-based genetic testing fails to identify a substantial proportion of at-risk individuals [25–30]. Furthermore, more comprehensive identification of variants in unselected individuals via genomic screening leads to risk management, can assist in syndrome-related diagnoses [26, 31], and may be cost-effective in some populations [32].

It is unclear whether these benefits of improved ascertainment of at-risk individuals or positive impacts on risk management are present when screening broader cohorts for ETS risk. Here, we describe an observational study examining a healthcare system that screens biobank participants for P/LP variants in genes associated with MEN 1, MEN 2, PGL/PCC, or VHL. In particular, we describe the personal and self-reported family history of syndrome-related features in patient-participants ascertained through the Geisinger MyCode® Community Health Initiative (MyCode), their risk management behaviors after receiving a result, and the rates of relevant post-disclosure diagnoses of neoplasms and other syndrome-related features.

## Methods

This study aims to provide critical insights needed to inform future genomic screening of unselected populations for ETS and support studies of longer-term clinical outcomes of genomic screening for ETS risk.

### MyCode Community Health Initiative

As described elsewhere, MyCode is a population health genomics project with more than 300,000 consented participants [33, 34]. The aim of the MyCode study is to provide participant data to enable translational research [33]. Any pediatric or adult Geisinger patient can voluntarily enroll in MyCode at primary care and specialty clinics throughout Geisinger regardless of their clinical history [26]. Those who enroll consent to health-based research including genomic analysis and during consent are informed that medically important genomic results will be reported to them [35]. MyCode participants overall have higher rates of self-reported White race and non-Hispanic ethnicity, older median age, and higher comorbidity index compared to the overall Geisinger population [26]. A subset of MyCode participants have undergone exome sequencing as part of the DiscovEHR collaboration with Regeneron Genetics Center [35, 36].

In 2013, the MyCode consent outlining participation in the research initiative was amended to enable the disclosure of actionable genomic findings to participants; individuals who enrolled prior to this date were invited to reconsent [33, 36]. In 2015, Geisinger began returning clinically actionable results to adult (≥ age 18) MyCode participants through the Genomic Screening and Counseling program (GSC) [37–39]. As part of GSC, variant call files generated through exome sequencing are analyzed for P/LP variants in actionable genes selected by MyCode leadership [26]. The MyCode gene list is periodically revised based on emerging evidence and, as of March 2022, includes 59 genes designated for assessment and return as secondary findings on indication-based testing by the American College of Medical Genetics and Genomics Secondary Findings V2.0 and *HFE* (OMIM 613609; only individuals homozygous for NM_000410.3:c.845G>A (p. Cys282Tyr)), associated with hereditary hemochromatosis [36, 40]. Copy number variants are not systematically examined in the current variant analysis pipeline. If confirmed and classified as P/LP by a CLIA-certified, clinical genetic testing laboratory, variants are disclosed to participants and their primary care providers (PCPs) and added to their electronic health record (EHR) problem list. PCPs are provided with brief “just in time” education along with the result notification that summarizes the condition, risks, and recommended management [38]. The GSC team attempts to reach all participants via three phone and/or EHR patient portal messages. Finally, all participants are sent a follow-up letter including details about the result and information for family members [38, 39]. GSC covers the cost of clinical confirmation of the variant, and all participants are offered complimentary genetic counseling following result disclosure that includes a discussion of the result, management recommendations, and implications for family members [38].

### Sample population

**Fig. 1:**
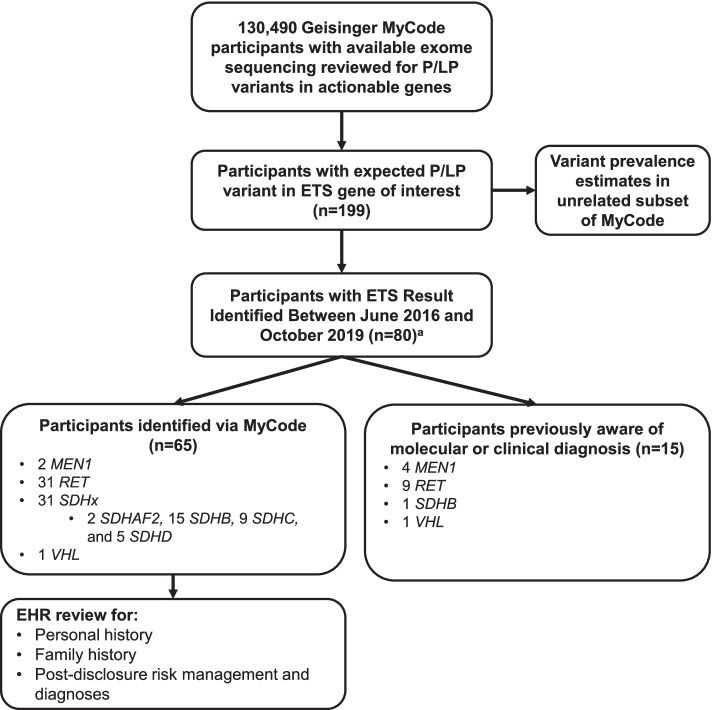
Sample population. ^a^119 participants with variants in genes of interest were excluded from further analysis for a variety of reasons including not being on a consent that allows result disclosure, being deceased at the time of result identification, and disclosure after the study period

As of July 2021, exome sequencing data from 130,490 adult Geisinger MyCode participants had been reviewed by the MyCode variant filtration and screening pipeline for P/LP sequence variants in genes designated as actionable (hereafter referred to as “participants with reviewed exome sequencing”) [36], including P/LP variants in *MEN1*, *RET*, *SDHAF2*, *SDHB*, *SDHC*, *SDHD*, and *VHL*. To allow for enough time post-disclosure for participants to have the opportunity to follow up on their result, we focus on individuals with a P/LP variant in *MEN1*, *RET*, an *SDHx* gene, or *VHL* disclosed between June 2016 and October 2019. Because this study aims to provide insights on screening broader, unselected populations for ETS, analyses further focus on the subset of participants without documentation of a genetic or clinical diagnosis consistent with the variant identified prior to MyCode result disclosure (hereafter referred to as “participants identified via MyCode”) (Fig. 1).

### Variant prevalence and genetic relatedness in Geisinger MyCode participants with reviewed exome sequencing

To report on variant prevalence in the unrelated adult MyCode participants with reviewed exome sequencing, genetic relatedness among those who were found to have a P/LP variant through the variant filtration and screening pipeline [32] was identified. First- and second-degree familial relationships were identified from exome sequence data using Pedigree Reconstruction and Identification of the Maximally Unrelated Set (PRIMUS) [41]. Code derived from the ukbtools [42] package was used to remove the fewest number of genetically related participants from the cohort of adult MyCode participants with reviewed exome sequencing to generate cohorts without first- and second-degree relatives. Two estimates of variant prevalence among unrelated participants were generated. The first estimate preferentially retained variant-positive participants among related pairs. For all pairs of first- and second-degree relatives in the adult MyCode cohort, the relative without a variant was removed. If both relatives had a variant, one was randomly removed. If neither relative had a variant, the one with the most relatives in the cohort was removed to minimize the number of participants dropped. If both variant-negative relatives had the same number of relatives in the cohort, one was randomly removed. Related pairs were re-calculated after each participant was dropped to minimize the total number of participants removed. The second estimate randomly retained one member of a related pair without regard to variant status. For all pairs of first- and second-degree relatives in the adult MyCode cohort, the one with the most relatives in the cohort was removed; otherwise, one was randomly removed. Related pairs were re-calculated after each participant was dropped to minimize the total number of participants removed.

### Outcomes in participants identified via MyCode

To characterize the personal and self-reported family history of ETS-associated features and clinical follow-up post-result disclosure in participants identified through MyCode, two independent reviews of the participants’ Geisinger EHRs were completed between May and July 2020 by certified genetic counselors (JMS, NMO, GMT). Reviewers followed a chart review abstraction guide to search the EHR and collect defined fields of interest, including personal and self-reported family history of syndrome-related findings and recommended surveillance and risk reduction activities based on the participant’s variant (Table 1). Personal and self-reported family history included history recorded at or prior to result disclosure. Reviewers examined the problem list and utilized the EHR search function to assess the personal history of syndrome-related features and post-disclosure diagnoses. Biochemical abnormalities such as hypercalcemia were not considered evidence of personal history given the nonspecific nature of these findings.

**Table 1:** Syndrome-related personal and self-reported family history and potential evidence of risk management assessed by manual EHR review^a^

Gene(s)	Personal history and self-reported family history	Potential evidence of risk management^**b**^
*MEN1* [1–3]	• Clinically identified P/LP *MEN1* variant • Multiple endocrine neoplasia type 1 • Wermer syndrome • Primary hyperparathyroidism • Parathyroid tumor • Pituitary adenoma • Zollinger-Ellison syndrome • Gastrinoma • Insulinoma • VIPoma • Glucagonoma • Pancreatic islet cell tumor • Well-differentiated endocrine tumors of the gastro-entero-pancreatic (GEP) tract • Carcinoid tumor • Adrenocortical tumor • Dermatologic manifestations o Facial angiofibroma o Collagenoma o Meningiomas o Ependymomas • Leiomyoma • Lipoma	**Appointments** • Genetics • Endocrinology • Otolaryngology • Gastroenterology **Surveillance** **Biochemical:** • Calcium • PTH, intact • 25-OH vitamin D • Prolactin • Insulin growth factor-1 • Gastrin • Fasting glucose • Insulin • Chromogranin A • Pancreatic polypeptide • Glucagon • Vasoactive intestinal peptide **Imaging:** • Head/brain MRI • Pituitary (Sella) MRI • Abdominal CT/MRI • Endoscopic ultrasound • Chest CT/MRI
*RET* [1, 4–6]	• Clinically detected P/LP *RET* variant • Multiple endocrine neoplasia type 2 • Medullary thyroid cancer • Pheochromocytoma • Parathyroid adenoma • Parathyroid hyperplasia • C-cell hyperplasia • Hirschsprung disease • Cutaneous lichen amyloidosis	**Appointments** • Genetics • Endocrinology • Otolaryngology **Surveillance** **Biochemical:** • Calcitonin • CEA • Calcium • PTH, intact • 25-OH vitamin D • Plasma-free metanephrines • 24-h urine fractionated metanephrines • Plasma-free catecholamines • 24-h urine fractionated catecholamines • Vanillylmandelic acid **Imaging:** • Thyroid ultrasound • Abdominal MRI/CT **Prophylactic surgery** • Thyroidectomy
*SDHx (SDHAF2, SDHB*, *SDHC*, *SDHD)* [1, 7–10]	• Clinically identified P/LP *SDHx* variant • Hereditary paraganglioma-pheochromocytoma syndrome • Paraganglioma • Pheochromocytoma • Gastrointestinal stromal tumor • Renal cell carcinoma	**Appointments** • Genetics • Otolaryngology • Endocrinology **Surveillance** **Biochemical:** • Plasma-free metanephrines • 24-h urine fractionated metanephrines • Plasma-free catecholamines • 24-h urine fractionated catecholamines • Vanillylmandelic acid • Dopamine and/or 3-methyoxytyramine **Imaging:** • Whole body CT/MRI ○ Head/neck CT or MRI ○ Abdomen/pelvis CT or MRI • Renal ultrasound
*VHL1* [1, 12, 13, 15–17]	• Clinically identified P/LP *VHL* variant • Von Hippel-Lindau syndrome • Renal cell carcinoma • Pheochromocytoma • Endolymphatic sac tumor • Hemangioblastoma (brain, spinal, retinal) • Renal cysts • Pancreatic cysts • Pancreatic neuroendocrine tumor • Epididymal and broad ligament cystadenomas	**Appointments** • Ophthalmology • Audiology • Otolaryngology • Urology • Endocrinology • Genetics **Surveillance** **Biochemical:** • Plasma-free metanephrines • 24-h urine fractionated metanephrines • Plasma-free catecholamines • 24-h urine fractionated catecholamines • Vanillylmandelic acid **Imaging:** • Abdominal ultrasound • Abdominal MRI **Exam:** • Audiology assessment • Dilated retinal exam

^a^Reviewers followed a chart review abstraction guide to search the EHR and collect defined fields of interest, including personal and self-reported family history (up to third-degree relatives) of syndrome-related findings and recommended surveillance and risk reduction activities based on the participant’s variant. This table represents an EHR search strategy. As such, synonyms and outdated diagnostic terms are included to ensure the EHR review was complete as possible. ^b^The risk management behavior list extends to activities that could be considered associated with the ETS variant, even if not the current standard of care for individuals with an ETS variant or if only considered in certain clinical scenarios, in an effort to accurately assess whether they may have been any interventions of relevance. This broad list was constructed by reviewing relevant guidelines and the literature [4, 5, 9, 13–20] and sought to recognize variation in practice, longer time since relevant guidelines were released, and a lack of consensus guidelines for all conditions

Participant-provided family history was captured from chart notes, the family history summary, and pedigree collected by GSC genetic counselors (if available). Reviewers used genetic relatedness data to attempt to determine if any self-reported family history of ETS-related features was secondary to MyCode result disclosure in the family member. ETS-related features reported in family members that were identified due to result disclosure via MyCode were not included in the analysis (e.g., if a family member was reported to have a *RET* variant by the participant, but genetic relatedness data enabled us to determine this result was identified via MyCode, this was not counted as syndrome-related family history).

Post-disclosure risk management was assessed by reviewing chart notes, relevant laboratory and imaging orders, and surgical history within the Geisinger EHR. To accurately assess whether post-disclosure interventions were associated with the ETS variant, the risk management list includes interventions that could be considered associated with the ETS variant, even if not reflected in the current standard of care for individuals with an ETS variant. This broad list was constructed by reviewing relevant guidelines and the literature [4, 5, 9, 13–20] and consulting with clinical colleagues caring for patients with ETS variants. The list sought to recognize variation in practice, evolving surveillance methods adopted since relevant guidelines were released, and a lack of consensus guidelines for some conditions. Reviewers analyzed chart notes and orders for biochemical, imaging, and surgical interventions to attempt to determine whether these risk management procedures were attributed to the disclosure of the genetic variant. All discrepancies between reviewers were resolved through joint review and consensus. Each reviewer collected and managed data using Research Electronic Data Capture (REDCap) tools hosted at Geisinger [43, 44].

Two independent reviewers (JMS and AHB) evaluated participants’ personal and self-reported family histories to determine if the participant met established referral guidelines for cancer predisposition assessment from the American College of Medical Genetics and Genomics and the National Society of Genetic Counselors [24] prior to result disclosure.

### Ethics

This work, including MyCode participation, result disclosure, and the research outlined, is approved by the Geisinger Institutional Review Board (IRB 2006-0258 and 2016-0229). Informed consent was obtained from all participants.

### Statistical methods

Descriptive statistics are summarized as median and interquartile range or frequency and percentage, as appropriate. Pearson’s chi-square and Wilcoxon rank sum tests were used to compare demographics of MyCode participants with a P/LP variant in a gene of interest disclosed before October 2019 to the remaining MyCode cohort with reviewed exome sequencing (*α* = 0.01 to account for multiple testing). To assess factors potentially associated with completion of a risk management behavior post-disclosure, such as age, sex, personal history, self-reported family history, and time since results, bivariate analyses were completed using Fisher’s exact test. The significance threshold was 0.01 to account for multiple testing. Analyses were primarily conducted using SAS (MPSAS v9.4 (SAS Institute Inc., Cary, NC)). Estimates of variant prevalence controlled for genetic relatedness were conducted using R version 4.0.1.

## Results

### Variant prevalence in Geisinger MyCode participants with reviewed exome sequencing

Potential P/LP variants in *MEN1*, *RET*, *SDHAF2*, *SDHB*, *SDHC*, *SDHD*, and *VHL* were identified in 199 of the 130,490 MyCode participants with reviewed exome sequencing data (equivalent to 1 P/LP variant per 656 participants). Since variants associated with ETS risk are often inherited, we removed genetically identified first- and second-degree relatives to obtain two estimates of variant prevalence in an unrelated subset of MyCode participants. When preferentially keeping variant-positive individuals among related pairs, 1 in 622 of unrelated MyCode participants were variant-positive (*n*=140/87166). When randomly removing a relative regardless of variant status, 1 in 751 (*n*=116/87201) participants were variant-positive. The true variant prevalence in this healthcare population likely lies between these two estimates.

### Characteristics of Geisinger MyCode participants with an ETS result identified between June 2016 and October 2019 (n=80) compared to remaining MyCode participants with reviewed exome sequence as of October 2019

Demographics of all participants who received an ETS-associated result between June 2016 and October 2019 (*n*=80) and the remaining MyCode participants whose exome sequence was reviewed for variant return as of October 2019 are summarized in Table 2; variant information is summarized in Table 3. Participants that received a variant of interest were 65% female, 99% self-reported their race as White, 99% self-reported non-Hispanic ethnicity, and they had a median age of 57 years at result disclosure (range 21–88 years). Seventy-six participants (95%) were alive at the time of initial data pull. No statistically significant differences between MyCode participants with an ETS result identified in the study period and the remaining MyCode participants without an ETS-associated variant as of October 2019 were identified.

**Table 2:** Demographics of Geisinger MyCode participants with an ETS result identified between June 2016 and October 2019 (*n*=80) by gene and compared to remaining MyCode cohort with reviewed exome sequencing

Demographic	***MEN1*** (***n***=6)	***RET*** (***n***=40)	***SDHx*** (***n***= 32)	***VHL*** (***n***=2)	Any variant associated with an endocrine tumor syndrome^**a**^ (***n***=80)	Remaining MyCode with reviewed exome sequencing^**b**^ (***n***=86,493)	***p***-value
**Median age (IQR)**							
Age at initial chart review/withdrawal	46.3 (46.3–55.7)	62.1 (41.5–72.5)	56.5 (46.9–68.1)	49.1 (52.0–54.9)	58.4 (42.1–70.2)	61.9 (47.3–73.1)	*p*=0.07
Age at result return	43.2 (42.0–52.1)	60.6 (40.9–71.1)	55.8 (45.2–66.0)	47.6 (50.8–53.9)	56.8 (41.4–68.0)	N/A	
**Sex**							
Male	16.7% (1)	47.5% (19)	21.9% (7)	50.0% (1)	35.0% (28)	38.6% (33379)	*p*=0.80
Female	83.3% (5)	52.5% (21)	78.1% (25)	50.0% (1)	65.0% (52)	61.4% (53109)	
Unknown	–	–	–	–	–	0.0% (5)	
**Race**							
White	100% (6)	100% (40)	96.9% (31)	100% (2)	98.7% (79)	97.6% (84387)	*p*=0.49
Black/African American	**–**	**–**	3.1% (1)	**–**	1.3% (1)	1.7% (1450)	
American Indian/Alaska Native	**–**	**–**	**–**	**–**	–	0.1% (109)	
Asian	**–**	**–**	**–**	**–**	–	0.3% (271)	
Native Hawaiian/Other Pacific Islander	**–**	**–**	**–**	**–**	–	0.1% (116)	
Unknown	**–**	**–**	**–**	**–**	–	0.2% (160)	
**Ethnicity**							
Not Hispanic or Latino	100% (6)	100% (40)	96.9% (31)	100% (2)	98.7% (79)	97.1% (83954)	*p*=0.80
Hispanic or Latino	**–**	**–**	3.1% (1)	**–**	1.3% (1)	1.7% (1480)	
Unknown	**–**	**–**	**–**	**–**	–	1.3% (1059)	
**Alive at initial data pull**	100% (6)	95.0% (38)	93.8% (30)	100% (2)	95% (76)	89.3% (77248)	*p*=0.10
**Smoking status**							
Current smoker	16.7% (1)	30.0% (12)	9.4% (3)	**–**	20.0% (16)	16.4% (14212)	*p*=0.39
Former smoker	33.3% (2)	35.0% (14)	40.6% (13)	50.0% (1)	37.5% (30)	35.9% (31083)	
Never smoker	50.0% (3)	35.0% (14)	50.0% (16)	50.0% (1)	42.5% (34)	47.3% (40916)	
Unknown	**–**	**–**	**–**	**–**	–	0.3% (282)	
**Time since results return (years; IQR)**	3.1 (2.9**–**3.5)	0.7 (0.6**–**2.9)	1.7 (0.7**–**2.7)	1.4 (0.8**–**1.9)	1.9 (0.6–2.9)	N/A	N/A
**Variant previously detected**							
Yes	50% (3)	22.5% (9)	3.1% (1)	**–**	16.3% (13)	N/A	N/A
No, but clinical diagnosis	16.7% (1)	**–**	**–**	50% (1)	2.5% (2)	N/A	
No	33% (2)	77.5% (31)	96.9% (31)	50% (1)	81.3% (65)	N/A	
**Number of families**							
Total	4	23	31	2	60	N/A	N/A
Previously clinically ascertained	3	4	0	1	8	N/A	

^a^Participants who received a result between June 2016 and October 2019; ^b^remaining MyCode participants whose exome sequence was screened for variant return as of October 2019

**Table 3:** Variants identified in Geisinger MyCode participants between June 2016 and October 2019 (*n*=80)

Condition	Gene	Variant (transcript:cDNA (protein))	ClinVar variant ID	Reported genotype/phenotype relationship	Participants identified via MyCode (***n***=65)	Participants previously aware of molecular or clinical diagnosis (***n***=15)
Multiple endocrine neoplasia type 1	*MEN1*	NM_130804.2:c.1267G>A (p.Asp423Asn)	16703	N/A	2	0
	*MEN1*	NM_130804.2:c.249_252del (p.Ile85SerfsTer33)	16693	N/A	0	2
	*MEN1*	NM_130804.2:c.307del (p.Leu103CysfsTer16)	200996	N/A	0	2
Multiple endocrine neoplasia type 2	*RET*	NM_020975.4:c.2671T>G (p.Ser891Ala)	13951	Moderate MTC risk, ~10% incidence of pheochromocytoma and hyperparathyroidism	26	6
	*RET*	NM_020975.4:c.2410G>A (p.Val804Met)	37102		4	1
	*RET*	NM_020975.4:c.1998G>T (p.Lys666Asn)	24932	Other variants impacting this codon classified as moderate MTC risk, ~10% incidence of pheochromocytoma and hyperparathyroidism	1	0
	*RET*	NM_020975.4:c.1859G>A (p.Cys620Tyr)	13916	Other variant(s) impacting this codon classified as moderate, ~10–30% incidence of pheochromocytoma, and ~10% incidence of pheochromocytoma and hyperparathyroidism	0	1
	*RET*	NM_020975.4:c.1858T>G (p.Cys620Gly)	24905		0	1
Hereditary paraganglioma and pheochromocytoma syndrome	*SDHAF2*	NM_017841.2:c.37-1G>C	806678	Parent of origin effect	1	0
	*SDHAF2*	NM_017841.2:c.305_306insA (p.Asn103GlufsTer4)	532508		1	0
	*SDHB*	NM_003000.2:c.343C>T (p.Arg115Ter)	197210	N/A	2	1
	*SDHB*	NM_003000.2:c.137G>A (p.Arg46Gln)	183793	N/A	2	0
	*SDHB*	NM_003000.2:c.286+2T>A	140773	N/A	2	0
	*SDHB*	NM_003000.2:c.380T>G (p.Ile127Ser)	183814	N/A	2	0
	*SDHB*	NM_003000.2:c.72+1G>T	142764	N/A	1	0
	*SDHB*	NM_003000.2:c.445_446insTATGG (p.Gln149LeufsTer11)	504902	N/A	1	0
	*SDHB*	NM_003000.2:c.491delA (p.Gln164ArgfsTer11)	528750	N/A	1	0
	*SDHB*	NM_003000.2:c.600G>T (p.Trp200Cys)	183747	N/A	1	0
	*SDHB*	NM_003000.2:c.688C>T (p.Arg230Cys)	185077	N/A	1	0
	*SDHB*	NM_003000.2:c.689G>T (p.Arg230Leu)	184933	N/A	1	0
	*SDHB*	NM_003000.2:c.725G>A (p.Arg242His)	12781	N/A	1	0
	*SDHC*	NM_003001.3:c.397C>T (p.Arg133Ter)	183753	N/A	5	0
	*SDHC*	NM_003001.3:c.43C>T (p.Arg15Ter)	41776	N/A	4	0
	*SDHD*	NM_003002.3:c.242C>T (p.Pro81Leu)	6896	Parent of origin effect	3	0
	*SDHD*	NM_003002.3:c.112C>T (p.Arg38Ter)	6893		1	0
	*SDHD*	NM_003002.3:c.53-1_53delGCinsTT	579812		1	0
Von Hippel-Lindau syndrome	*VHL*	NM_000551.3:c.562C>G (p.Leu181Val)	2225	VHL type 2C	1	0
	*VHL*	NM_000551.3:c.292T>C (p.Tyr98His)	2223	VHL Type 2A	0	1

### Characteristics of participants identified via MyCode (*n*=65)

The 65 participants identified via MyCode were 66% female (*n*=43), 98.5% self-reported their race as White (*n*=64), 98.5% self-reported non-Hispanic ethnicity (*n*=64), and they had a median age of 56.2 years at result disclosure (range 20.8–88.0 years). All participants were alive at the time of result disclosure. Three participants passed away between result disclosure and initial data pull; the cause of death was available for two participants and was unrelated to the genetic result. The median length of time since result disclosure was 0.7 years (range 0.1–4.0 years). Using genetic relatedness data, these 65 individuals are from 52 families — one *MEN1*, 19 *RET*, 31 *SDHx* (2 *SDHAF2*, 15 *SDHB*, 9 *SDHC*, and 5 *SDHD*), and one *VHL* family*.*

### Personal and self-reported family history in participants identified via MyCode (*n*=65)

Of the 65 participants identified via MyCode, 19 (29%) had a personal and/or self-reported family history consistent with their genomic result documented in the EHR at the time or result disclosure (Table 4, Additional file 1: Table S1). Five individuals (8%) from five families, all of whom received an *SDHx* variant (4 *SDHB*, 1 *SDHD*), had a personal history (2 with a GIST (2 *SDHB*), 2 with a paraganglioma (1 *SDHD*, 1 *SDHB*), and 1 with paragangliomas and renal cell carcinoma (1 *SDHB*)).

**Table 4:** Frequency of personal and self-reported family history^a^ of syndrome-related phenotypes pre-result disclosure in participants identified via MyCode

Gene	Personal history of ETS-associated feature(s)	Self-reported family history of ETS-associated feature(s)	Met genetics referral criteria
**All** ***n*=65**	8% (*n*=5/65)	25% (*n*=16/65)	11% (*n*=7/65)
***MEN1*** ***n=2***	0% (*n*=0/2)	100% (*n*=2/2)	0% (*n*=0/2)
***RET*** ***n=31***	0% (*n*=0/31)	26% (*n*=8/31)	10% (*n*=3/31)
***VHL*** ***n=1***	0% (*n*=0/1)	0% (*n*=0/1)	0% (*n*=0/1)
***SDHx*** ***n=31***	16% (*n*=5/31)	19% (*n*=6/31)	13% (*n*=4/31)
***SDHAF2*** ***n*=2**	0% (*n*=0/2)	50% (*n*=1/2)	0% (*n*=0/2)
***SDHB*** ***n=15***	27% (*n*=4/15)	27% (*n*=4/15)	20% (*n*=3/15)
***SDHC*** ***n=9***	0% (*n*=0/9)	11% (*n*=1/9)	0% (*n*=0/9)
***SDHD*** ***n=5***	20% (*n*=1/5)	0% (*n*=0/5)	20% (*n*=1/5)

^a^Personal and self-reported family history of syndrome-related findings are noted in Table 1

Sixteen individuals (25%) from ten families (19%) had a self-reported family history consistent with their genomic result. Two individuals with an *MEN1* variant from one family had a self-reported family history of a pancreatic neuroendocrine tumor. Six individuals with an *SDHx* variant from six families had a self-reported family history of *SDHx*-related features (4 *SDHB*, 1 *SDHC*, 1 *SDHAF2*); one had a self-reported family history of a pheochromocytoma and renal cell carcinoma (1 SDHB) and the other five had a self-reported family history of renal cell carcinoma (3 *SDHB*, 1 *SDHC*, 1 *SDHAF2*). Eight participants with a *RET* variant from three families had a self-reported family history consistent with the variant, including a family history of a medullary thyroid cancer, unknown thyroid cancer, and/or clinically identified *RET* variant.

Of the nineteen participants with a personal and/or self-reported family history consistent with their genomic result, only seven (37%) met cancer genetics referral guidelines [24] prior to result disclosure based on their personal and/or self-reported family history (Additional file 1: Table S2). All who met referral criteria pre-disclosure were identified to have a *RET* (*n*=3) or *SDHx* variant (3 *SDHB*, 1 *SDHD*); three had a personal history of paragangliomas (2 *SDHB*, 1 *SDHD*) and four had a self-reported family history warranting referral — one with an *SDHB* variant who reported a first-degree relative with renal cell carcinoma before age 50 and three with *RET* variants who reported that a family member had been clinically identified to carry the variant prior to result disclosure. None of the seven participants meeting referral guidelines [24] had been referred to a genetics provider prior to result disclosure.

### Post-disclosure management in participants identified via MyCode (n=65)

Following result disclosure, 35 participants (54%) identified via MyCode met with a genetics provider (geneticist and/or genetic counselor) through GSC, 20 (31%) discussed the result with a PCP, and 27 (42%) discussed the result with a relevant specialist provider (e.g., endocrinologist, otolaryngologist). Twenty-five participants (38%) met with multiple providers to discuss their genomic results. In total, 55% (*n*=36/65) of participants completed a recommended risk management behavior post-disclosure (Table 5), including 52% (*n*=34/65) of participants that completed biochemical surveillance (median 54 days post-result disclosure, IQR 33–127 days) and 48% (*n*=31/65) that completed imaging surveillance (median 80 days post-result disclosure, IQR 47–200 days). Of the 31 individuals with a *RET* variant, nine (29%) underwent thyroidectomy (median 133 days post-result disclosure, IQR 85–172, results below). In looking at overall follow-up rates by condition, both participants with an *MEN1* variant, the participant with a *VHL* variant, 52% of participants with an *SDHx* variant (*n*=16/31), and 55% of participants with a *RET* variant (*n*=17/31) completed at least one risk management behavior. From the EHR review, 78% (*n*=28/36) of participants that completed a risk management behavior had that behavior clearly attributed to the genetic result. From bivariate analysis, meeting with a genetics provider to discuss the result and seeing a specialist provider were significantly associated with the performance of a risk-related management behavior post-disclosure (Table 5). Sex, age, time since results, family history, personal history, and PCP follow-up were not associated with the performance of a risk-related management behavior post-disclosure (Table 5).

**Table 5:** Association of participant characteristics and performance of risk management behavior(s)^a^ post-disclosure in participants identified via MyCode

Variable	Participants identified via MyCode (***n***=65)	Post-disclosure risk management behavior (*n*=36)	No post-disclosure risk management behavior (***n***=29)	Odds ratio (95% confidence interval), ***p***-value
Female sex	43/65 (66.2%)	27/36 (75%)	16/29 (55.2%)	0.71 (0.52–2.01), 0.09
Median age at result return — years (IQR)	56.2 (30.2)	57.2 (29.0)	55.4 (28.9)	1.3 (0.18–8.15), 0.68
Median time since results — years (IQR)	0.7 (2.2)	1.1 (2.2)	0.7 (1.4)	6.2 (0.96–40.1), 0.38
Personal history	5/65 (7.7%)	4/36 (11.1%)	1/29 (3.4%)	1.76 (0.31–9.86), 0.25
Self-reported family history	16/65 (24.6%)	12/36 (33.3%)	4/29 (13.7%)	3.83 (0.97–15.2), 0.038
Genetics follow-up	35/65 (53.8%)	29/36 (80.6%)	6/29 (20.7%)	9.7 (3.02–30.9), <0.0001*
Specialist follow-up	27/65 (41.5%)	26/36 (72.2%)	1/29 (3.4%)	50 (6.1–411.1), <0.0001*
PCP follow-up	20/65 (30.8%)	15/36 (41.7%)	5/29 (17.2%)	5.9 (1.5–23.1), 0.0339

^a^Potential risk management behaviors are noted in Table 1. *denotes statistical significance

### Post-disclosure diagnoses in participants identified via MyCode (*n*=65)

Following result disclosure, 11 participants (17%) from six families received a syndrome-related diagnosis consistent with the variant identified. Both individuals with *MEN1* pathogenic variants were diagnosed with MEN 1-related features, one with primary hyperparathyroidism and a leiomyoma and the second with primary hyperparathyroidism, a leiomyoma, and a pituitary adenoma. One individual with an *SDHB* pathogenic variant (3% of participants with *SDHx* result and 7% with *SDHB* result via GSC) was diagnosed with a left head and neck paraganglioma post-result disclosure. Finally, eight of the nine individuals that received a *RET* pathogenic result and underwent thyroidectomy post-disclosure were diagnosed with medullary thyroid cancer (26% of participants with *RET* result via GSC, 89% that had thyroidectomy, median age at diagnosis 58.7 (range 33.6–72.9) years). The ninth participant was diagnosed with a papillary carcinoma, follicular variant. No participants that received a post-disclosure diagnosis had a personal history of syndrome-related diagnoses prior to receiving their results. Eight had a self-reported family history of syndrome-related features documented, and two met referral criteria for a cancer genetics evaluation at or before the result disclosure.

## Discussion

This study reports on the experience screening a healthcare system population for genetic variants associated with ETS risk. We found that ETS are much more common than previously reported, genomic screening for ETS can ascertain at-risk individuals who should have already been ascertained but were not, as well as those who would not have otherwise come to clinical attention, and genomic screening facilitated diagnoses of endocrine neoplasms in several participants. Yet, further research is necessary to address key outstanding questions on the clinical utility of genomic screening for ETS.

By screening an unselected healthcare system cohort for P/LP variants in ETS genes, we found a considerably higher rate of ETS — 1:622 to 1:751 — than is commonly reported. Although this variant prevalence is only an estimate due to conservative pipeline filtering [36], this estimate is over eleven times greater than the previously reported cumulative disease prevalence for MEN 1, MEN 2, PGL/PCC, and VHL of approximately 1:8500 [2]. These results are consistent with a recent abstract summarizing a study of *RET* variants in a healthcare-based biobank that found such variants are more common than anticipated based on disease prevalence [45]. The high variant prevalence reported here is, in part, due to the genotype-first approach undertaken in this study and the expected reduced penetrance of some variants (e.g., penetrance of maternally inherited *SDHAF2* and *SDHD* variants). However, these data suggest that variants associated with ETS risk are more common than previously reported. Such data inform future population screening efforts by providing an estimated number of returnable variants in a healthcare-based cohort. These data are needed to contribute to future studies (e.g., All of Us [45] and Genomics England [46]) performing genomic screening as well as cost-effectiveness analyses that will be necessary to further assess the feasibility of genomic screening for these conditions [47]. Furthermore, although some variants are expected to have reduced penetrance, such estimates are based on clinical cohorts that tend to overestimate prevalence based on the ascertainment of individuals with a personal or family history of disease. Identifying variant prevalence and exploring personal and family history data in broader cohorts such as this will be needed to expand our understanding of penetrance and specific genotype/phenotype correlations.

This study also provides data regarding how broader screening efforts could increase the identification of at-risk individuals and suggests how such screening could augment current clinical care. Our data provide evidence of under-ascertainment of ETS in current clinical practice in two ways. First, despite having a personal and/or self-reported family history consistent with established cancer genetics referral guidelines [24], none of the seven MyCode-identified participants who met referral criteria had been evaluated clinically pre-disclosure, suggesting a need to more effectively identify patients who meet current referral criteria. Provider education and clinical decision support in the EHR could improve the clinical ascertainment of these at-risk participants. Population screening should not replace clinical evaluations of those meeting referral guidelines [47], but these data suggest such screening could capture those who have been missed by current practices. Second, referral guidelines are not sufficiently sensitive to identify all individuals with ETS risk. The majority of participants with a variant associated with ETS risk (89%, *n*=58/65) did not meet genetics referral criteria pre-disclosure. These data suggest that, even if referral criteria were appropriately applied, individuals with P/LP ETS variants would be missed in current clinical practice. Moreover, the distribution across genes differs in the participants who were previously clinically identified compared to those identified via MyCode (Additional file 1: Table S1), suggesting that under-ascertainment via current clinical practice might differ by gene (e.g., increased under-ascertainment of *SDHx* compared to *MEN1*). Similar findings of under-ascertainment in current practice have been illustrated in other hereditary cancer syndromes and actionable conditions [28, 29]. This suggests that broader testing strategies, such as population genomic screening, could provide an approach to better identify at-risk individuals.

Improved identification of at-risk individuals will not improve health outcomes if no change in health behavior follows disclosure. This study provides further evidence that genomic screening can prompt changes to clinical care. The majority (55%) of individuals who were not previously aware of their result completed at least one associated risk management behavior after disclosure (biochemical/imaging surveillance or surgical intervention). The number of individuals who sought additional care post-disclosure is similar to those with other actionable genetic risks including variants associated with hereditary breast and ovarian cancer syndrome, Lynch syndrome, and familial hypercholesterolemia [26]. Participants who saw a genetics provider and those who met with a specialist were more likely to complete recommended risk management compared to those who did not meet with these providers. It is not clear whether genetic counselors and/or specialists facilitated the performance of recommended risk management or whether individuals who chose to meet with a genetic counselor and/or specialist are more engaged in health care generally, or more likely to complete recommended follow-up due to other factors. Follow-up with a PCP was not associated with the performance of risk-related management behaviors post-disclosure. With the EHR data available, it is unclear whether this is because PCPs did not recommend risk-related management or if patients did not act on such recommendations. Future studies should continue to explore facilitators and barriers to patient follow-up to determine how best to facilitate the complementary roles PCPs, genetic counselors, endocrinologists, and other specialists can play in communicating and coordinating risk management for individuals with a P/LP ETS variant.

The diagnosis of MTC in eight of nine participants who underwent post-disclosure thyroidectomy illustrates the promise of population screening for genetic disease risk to inform care and enable diagnoses. It may be that there is particular utility in screening populations for *RET* variants and that additional interventions to encourage thyroidectomies among participants with a *RET* variant are indicated. Or, it may be that these cases represent lead time bias, over-diagnosis, and over-treatment. Resolving this question will require larger, longitudinal studies designed to elucidate the impact of population genetic screening on morbidity and mortality. Such studies can also investigate whether to alter risk management recommendations for individuals ascertained from population-based cohorts [47]. For example, if further studies support that thyroidectomy in patients with *RET* variants identified via genomic screening leads to over-diagnosis, perhaps a stepped approach to risk management in adults that begins with calcitonin screening and imaging to determine whether to proceed to thyroidectomy in asymptomatic individuals with *RET* variants could be considered. Biochemical screening may offer a less burdensome management behavior that can enable earlier diagnoses in those identified to have genomic ETS risk via broader screening. Further studies will be needed to resolve this question, explore additional factors that might be important when determining individualized risk management (e.g., age, specific variant detected), and generate evidence to determine the appropriateness of altering care in those receiving *RET* results in broader populations. Until such studies are completed, care based on current guidelines represents the conservative approach.

Two limitations of this study are the small sample size and short follow-up time, which limit our ability to determine factors associated with post-disclosure risk management through bivariate analyses, our capacity to use more sophisticated modeling, and our ability to evaluate long-term outcomes of disclosure and follow-up care. The small number of participants receiving *MEN1* and *VHL* variants limits our capacity to draw conclusions about the disclosure of variants in those genes.

Furthermore, personal history, self-reported family history, and post-disclosure risk management relied on data available in the Geisinger EHR which has its limitations as a data source. Personal history of ETS based on the EHR might not include all such diagnoses if a patient receives care outside of the system. Self-reported family history might be incomplete for some patients since capture and recording of these data vary (e.g., 21% of participants had a pedigree while the remaining only had family history data in the EHR). As such, relying on EHR data might lead to underestimates of relevant personal and family history [29]. Additionally, post-disclosure risk management relied on documentation of management in the EHR which, again, fails to capture care that occurs outside of the system. This could lead to an underestimate of the impact of these results on care and diagnoses. Finally, while we attempted to determine if the risk management behaviors were clearly attributed to the disclosure of the genetic variant identified, the indication for surveillance was not always clearly documented in the EHR. Future studies that include EHR data as well as other data sources, including patient-reported care information could further determine the clinical impact of these genetic results.

Other genes (e.g., *SDHA*, *MAX*, *TMEM127*, *PRKAR1A*) associated with ETS risk were not included in this analysis since they were not being disclosed to MyCode participants at the time of the study. Additionally, genes that have clinical presentation and management that are not primarily endocrine tumor syndrome focused (e.g., *PTEN* with risks for thyroid cancer as well as risks for neurodevelopmental disorders, colorectal, breast, and endometrial cancers) were also excluded.

It is unclear if these findings will be generalizable to more racially and ethnically diverse cohorts, to other healthcare systems, or to the general population. Additional studies, including those with longer follow-up, those that compare rates of phenotypes to variant-negative and clinically ascertained individuals, and those that combine data from multiple, diverse cohorts, are needed to explore P/LP variant prevalence and penetrance in unselected cohorts, clinical utility and outcomes of genomic screening, cost-effectiveness of result disclosure, and care, and determine factors that are associated with post-disclosure risk management behaviors. Future studies will also be needed to examine the de novo rate and whether parent-of-origin effects are seen in genomic screening cohorts.

## Conclusion

Despite the need for additional studies to fully understand the clinical utility and outcomes of genomic screening for ETS, this work demonstrates that screening healthcare populations can enable the detection of individuals at genetic risk for ETS, lead to uptake of risk management, and facilitate relevant clinical diagnoses.

## Supplementary Information

**Additional file 1: Table S1. **Details of Participants Identified Via MyCode and Participants Previously Clinically Identified Stratified by Variant. *Families based on genetic-relatedness data. **Table S2.** Participants Identified Via MyCode Meeting Cancer Genetics Referral Criteria. ^a^The American College of Medical Genetics and Genomics and National Society of Genetic Counselors publication summarizing cancer genetics referral guidelines states that a referral for cancer genetics should be considered for probands with a personal history of renal cancer before age 50 or a first degree relative with such a history. While SDHB is not listed in the example conditions that could be associated with such a history in those guidelines, SDHB has been associated with renal cancer risk since the 2000s and, as such, has been on renal cancer genetic testing panels for some time. As such, the genetic counselor reviewers counted this as meeting referral criteria for cancer genetics evaluation.

## Data Availability

Some or all datasets generated during and/or analyzed during the current study are not publicly available but are available from the corresponding author on reasonable request.

## Abbreviations

EHR: Electronic health record
ETS: Endocrine tumor syndrome
GIST: Gastrointestinal stromal tumor
GSC: Genomic screening and counseling program
MEN 1: Multiple endocrine neoplasia type 1
MEN 2: Multiple endocrine neoplasia type 2
P/LP: Pathogenic/likely pathogenic
PCP: Primary care provider
PGL/PCC: Hereditary paraganglioma and pheochromocytoma syndrome
PRIMUS: Pedigree reconstruction and identification of the maximally unrelated set
*SDHx*: *SDHB*, *SDHC*, *SDHD*, *SDHAF2*
VHL: Von Hippel-Lindau syndrome
